# Epigenetic Modifications Induced by Olive Oil and Its Phenolic Compounds: A Systematic Review

**DOI:** 10.3390/molecules26020273

**Published:** 2021-01-07

**Authors:** Roberto Fabiani, Nicolò Vella, Patrizia Rosignoli

**Affiliations:** Department of Chemistry, Biology, and Biotechnology, University of Perugia, Via del Giochetto, 06126 Perugia, Italy; vella.nicola93@gmail.com (N.V.); patrizia.rosignoli@unipg.it (P.R.)

**Keywords:** extra virgin olive oil, secoiridoids, phenolic compounds, epigenetics, DNA methylation, histone modification, miRNA, anti-cancer, anti-inflammatory

## Abstract

Many studies demonstrated that olive oil (especially extra virgin olive oil: EVOO) phenolic compounds are bioactive molecules with anti-cancer, anti-inflammatory, anti-aging and neuroprotective activities. These effects have been recently attributed to the ability of these compounds to induce epigenetics modifications such as miRNAs expression, DNA methylation and histone modifications. In this study, we systematically review and discuss, following the PRISMA statements, the epigenetic modifications induced by EVOO and its phenols in different experimental systems. At the end of literature search through “PubMed”, “Web of Science” and “Scopus”, 43 studies were selected.Among them, 22 studies reported data on miRNAs, 15 on DNA methylation and 13 on histone modification. Most of the “epigenomic” changes observed in response to olive oil phenols’ exposure were mechanistically associated with the cancer preventive and anti-inflammatory effects. In many cases, the epigenetics effects regarding the DNA methylation were demonstrated for olive oil but without any indication regarding the presence or not of phenols. Overall, the findings of the present systematic review may have important implications for understanding the epigenetic mechanisms behind the health effects of olive oil. However, generally no direct evidence was provided for the causal relationships between epigenetics modification and EVOO health related effects. Further studies are necessary to demonstrate the real physiological consequences of the epigenetics modification induced by EVOO and its phenolic compounds.

## 1. Introduction

From the first evidence from the early 1960s suggesting a link between the eating habits of people living around the Mediterranean area and the low cardiovascular mortality, the Mediterranean diet (MD) has been the subject of countless studies aimed at investigating its potential preventive properties against different chronic-degenerative diseases [1]. Most of the epidemiological prospective cohort studies have reported an inverse association between adherence to MD and risk of cardiovascular disease, type 2 diabetes, cancer, and cognitive-related disorders [2]. Furthermore, MD adherence was inversely associated with the risk of all-cause mortality, especially in the Mediterranean regions [3]. Accordingly, in 2013, the UNESCO inscribed MD on the representative list of the Intangible Cultural Heritage of Humanity (https://ich.unesco.org/en/RL/mediterranean-diet-00884). Among other dietary characteristics, the main peculiarity of the MD is that olive oil is the primary lipids’ source. As suggested by many investigators, most of the MD human health promotion effects can be attributed to olive oil, specifically to “extra virgin olive oil” (EVOO). In the last few years, the health properties of EVOO have been deeply investigated and efficiently summarized in many excellent reviews [4,5,6].

The health promoting properties of EVOO have been correlated to its peculiar chemical composition [7]. EVOO contains both major (more than 98% of the total oil weight) and minor components. The major components include triglycerides and other fatty acids derivatives containing mainly monounsaturated fatty acids, in particular oleic acid (up to 83% of the total fatty acid content). Minor components are represented by more than 230 different chemicals including aliphatic and triterpenic alcohols, phytosterols, hydrocarbons, tocopherols, volatile compounds and polyphenols [8]. Although in the past the health effects of EVOO have been mainly attributed to oleic acid, more recently attention has been paid to phenolic compounds [9]. This class of bioactive compounds includes phenolic acids, phenolic alcohols, flavonoids, secoiridoids, and lignans. Among the phenolic alcohols, hydroxytyrosol (3,4-dihydroxyphenylethanol: 3,4-DHPEA) and tyrosol (p-hydroxyphenylethanol: *p*-HPEA) are of particular interest since they are abundantly and exclusively present in EVOO. They can be present as both free compounds and linked to either elenolic acid (EA) or its dialdehydic form (EDA), giving rise to the following secoiridoid derivatives: 3,4-DHPEA-EA (oleuropein aglycon), *p*-HPEA-EA (ligstroside aglycon), 3,4-DHPEA-EDA (oleacein), *p*-HPEA-EDA (oleocanthal) and oleuropein [10,11]. In addition to their potent anti-oxidant properties, these compounds have shown many additional biological activities both in vitro and in vivo systems. Indeed, several controlled human intervention trials have now confirmed that rich-polyphenols EVOO is able to improve some cardiovascular risk factors and prevent cardiovascular events [12]. Nevertheless, many studies have clearly demonstrated that EVOO phenolic compounds possess many other biological functions that can be correlated to their anti-cancer [11,13], anti-inflammatory, anti-aging, and neuroprotective activities [14,15]. Most of these effects have been related to the ability of phenols to control pathways of cell signaling, to modulate transcription factors activity, and to influence gene expression. Nutrigenomic properties of EVOO and its phenolic compounds have been recently reviewed [16].

Epigenetics is defined as reversible heritable changes, occurring without alteration of DNA sequence, which are able to regulate the gene expression. Three main related epigenetic mechanisms have been so far described: DNA methylation, histone modifications (acetylation and methylation), and post transcriptional gene regulation by non-coding microRNAs (miRNAs) [17]. The accurate regulation of the “epigenome” determines whether, when, and where a gene is either silenced or expressed. Disruption of epigenetic mechanisms has been correlated to chronic diseases upset, particularly cancer [18]. For instance, miRNAs may act as both oncogenes and tumor-suppressors, depending upon the inhibition of target genes [19]. Very interesting, being reversible, these processes may be influenced by dietary and environmental factors. Indeed, nutrition-induced epigenetic variation may occur throughout the life course [20].

In the last few years, many studies have investigated whether the bioactivity of EVOO and its phenols could be mediated by epigenetic mechanisms. However, up to now, no systematic revision has been published focusing on the possible effects of these compounds on miRNAs expression, modulation of DNA methylation, and histone modification. Therefore, the aim of this study was to systematically review and discuss the all literature data in which epigenetic effects have been demonstrated to be induced by both EVOO and its phenolic compounds. Our findings may have important implications for understanding the epigenetic mechanisms behind the health effects of olive oil.

## 2. Results and Discussion

**Figure 1:**
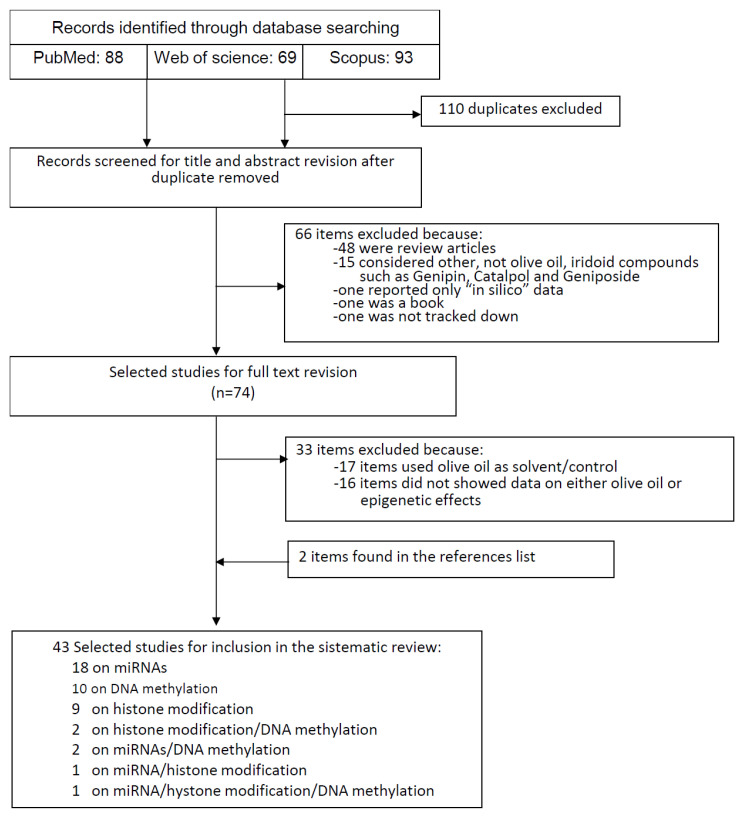
Flow diagram of study selection process.

From the primary literature research through PubMed (*n* = 88), Web of Science (*n* = 69) and Scopus (*n* = 93) databases and after removing duplicate (*n* = 110), 140 records were identified for title and abstract revision (Figure 1). From them, 66 items were excluded for the following reasons: 48 were review articles, 15 items considered other iridoid compounds such as Genipin, Catalpol and Geniposide not present in olive oil, one article reported only “in silico” data, one was a book, and one was not tracked down. The full text revision of the 74 remaining articles resulted in a further 33 items excluded as follows: 17 used olive oil as either solvent or control, and 16 items did not show data on either olive oil or epigenetic effects. In addition, two more articles, found in the references list of previous items, were included. Therefore, at the end of the selection process, 43 studies were included in the systematic review (Figure 1). Among them, 18 reported data on miRNAs [21,22,23,24,25,26,27,28,29,30,31,32,33,34,35,36,37,38], 10 on DNA methylation [39,40,41,42,43,44,45,46,47,48], 9 on histone modification [49,50,51,52,53,54,55,56,57], 2 on histone modification/DNA methylation [58,59], 2 on miRNAs/DNA methylation [60,61] one each on miRNA/histone modification [62] and on miRNA/histone modification/DNA methylation [63] (Figure 1).

### 2.1. miRNAs

The characteristics of the 22 selected studies investigating the effects of olive oil and its phenolic compounds on miRNA expression are shown in Table S1. Sixteen studies reported data in vitro on different cell systems [21,22,24,26,27,28,30,31,32,34,35,36,37,38,60,63], 9 studies showed results on animal models [23,24,29,30,33,60,61,62] and 2 studies reported results of human intervention trials [24,25]. Seven studies investigated the effects of hydroxytyrosol [24,27,28,33,35,36,62], 5 articles reported data on oleuropein [26,28,30,31,34], 5 studies were carried out with olive oil [23,25,29,60,61] and 2 studies with phenolic extracts obtained from *Olea europaea* leaves [21,22]. Two investigations reported data on oleacein [37,63] and one study each investigated the effect of hydroxytyrosol-oleate [32], hydroxytyrosol-3-*O*-sulphate [38] and oleocanthal [37].

#### 2.1.1. Anti-Cancer Effects

The anti-cancer properties of olive oil and its phenols have been widely investigated in several in vitro and in vivo systems. Secoiridoids and their derivatives have shown anti-proliferative, pro-apoptotic and pro-differentiation activities on different tumour cells. One molecular mechanism involved in these effects could be the modulation of miRNAs expression. Indeed, “oncomirs” is the name attributed to miRNAs having a role in the carcinogenic process, which may act as both tumour suppressors and oncogenes [19]. In this systematic review, we found that out of the 22 selected studies, 10 articles have correlated the up or down expression of different miRNA with an anti-cancer activity of phenols [21,22,25,26,30,31,34,60,61,63]. The first study, aimed to investigate the effect of olive oil phenols on miRNAs, was carried out using a phenolic extract obtained from leaves of *Olea europaea* (OLE). This phenolic extract, which was particularly rich in oleuropein, was tested on glioblastoma (GBM) cells [21]. Out of the 40 miRNAs screened, several were demonstrated to be either up- or down- regulated by OLE on both GBM cell lines [21] and primary stem-like GBM cells [22]. Interestingly, co-treatment of cells with one the most commonly used cytostatic drug Temozolomide (TMZ) caused a synergistic effect with OLE on the reduction of cell proliferation and induction of apoptosis. In addition, co-treatment changed the expression profile of a subset of the miRNAs. To identify differentially expressed miRNA target genes related to cell cycle progression and apoptosis pathways, bioinformatics analysis was used [21]. Some of these important genes, such as TP53 (tumour protein p53), OCT-4 (POU class 5 homeobox 1), SOX2 (SRY (sex determining region Y)-box 2), BCL2 (B-cell CLL/lymphoma 2) and c-myc were experimentally demonstrated to be down regulated by OLE in GBM stem-like cells [22]. The same experimental system was used to investigate the effect of pure oleuropein on miRNA expression [34]. The effects were sometime different compared to OLE, in particular regarding the expression of let-7d which was not significantly affected by OLE (< 2-fold), while it was dramatically increased by oleuropein (125 and 263-fold at 277.5 and 555 µM oleuropein concentrations, respectively) [34]. Some differences were also observed when the cells were co-exposed to oleuropein together with TMZ [34]. These results suggest that the complex olive leaves phenolic extract contains, in addition to oleuropein, other anticancer compounds acting with different molecular mechanisms. Further investigations are necessary to clarify this point, since no other studies have reported the effect of olive oil phenolic extracts on miRNA expression.

Instead, the anti-cancer properties of oleuropein in relation to the modulation of miRNA expression were investigated both in vitro on HNE1 and HONE1 nasopharyngeal carcinoma (NPC) cells [26], Caov3 and Skov3 ovarian cancer cells [30] and MCF-7 breast cancer cells [32], and in vivo on xenograft mouse models [26,30]. It was found that oleuropein is able to promote radiation sensitivity in NPC and ovarian cancer cells both in vitro and in vivo through the modulation of miRNA expressions [26,30]. In NPC cells, oleuropein reduced the DNA damage-regulated protein (PDRG1) expression via upregulation of miRNA-519d. This effect was mediated by downregulation of hypoxia-inducible factor-1α (HIF1α) expression and inhibition of its binding to the promoter of miRNA-519d [26]. In ovarian cancer cells oleuropein, by upregulation of miRNA-299, suppressed heparanase (HPSE1) expression leading to an increment of radiation sensitivity in these cells [30]. On the other hand, in MCF7 breast cancer cells, oleuropein repressed the expression of master “oncomirs” miRNA-21 and miRNA-155 [31]. This effect was associated with a reduction of viability and migration, induction of apoptosis, and upregulation of mRNA of some genes in cancer cells. However, no causality between miRNA effects and anti-cancer activity of oleuropein in this cellular system was investigated [31]. Different from oleuropein, a structurally similar molecule oleacein triggered upregulation of tumour suppressive miRNAs, including miRNA-29b and miRNA-22 in a human multiple myeloma (MM) cell line JJN3 [63]. This effect was associated with anti-cancer effects on MM cells (inhibition of proliferation and cell cycle, induction of apoptosis) and inhibition of the oncogenic transcription factor Sp1. Nevertheless, also in this case, causality between these effects was not investigated [63]. Another compound of particular relevance in this contest is hydroxytyrosol, the foremost and most widely investigated phenolic component of EVOO. It is present in the molecular structure of oleuropein and oleacein, and it is released during digestion of these secoiridoids. It is curious to note that no study has investigated whether the chemopreventive properties of hydroxytyrosol on cancer cells could be correlated to and/or mediated by alteration of miRNA expression.

Three studies have investigated the effect of EVOO on miRNA expression in relation with its anti-cancer effects. From an in vitro experiment on CaCo-2 colon carcinoma cells, a phenolic extract obtained from EVOO was found to inhibit the proliferation and upregulate the expression of the tumour suppressor gene type 1 cannabinoid receptor (CB1) [60]. This effect was confirmed in vivo on rats treated with EVOO (containing a significant amount of phenols: 320 mg/Kg) both with a single dose and with 10-day administration. Successively, EVOO was tested to reveal its effect on the expression of four miRNAs known to be involved in the pathogenesis of colorectal cancer. The expression of two of them, miRNA23a and miRNA301a, was found to be selectively reduced after either single or 10-day administration of EVOO [60]. Since the control group of rats received water, we do not know which component of EVOO was responsible for the effects observed. Similarly, in a colon carcinogenic model of dimethylhydrazine (DMH)-treated rats, an evident anti-cancer effect of EVOO was found in terms of a reduced tumour incidence, multiplicity and volume [61]. These effects were associated with a reduction of inflammatory markers (see below) and to an increment of apoptotic markers (caspase-3 and caspase-9). At the same time, an upregulation of “tumour suppressors” miRNA-143 and miRNA-145 was observed together with a heavy hypermethylation of their promoter regions [61]. However, no causality was demonstrated and no conclusion on the role played by phenols can be inferred since no appropriate control was available and no data on the content of phenols present in the EVOO was provided [61]. Further observations of this article on DNA methylation changes induced by EVOO on Cnr1 gene promoter will be discussed below. Finally, a human trial was aimed to investigate the effects of EVOO on the miRNome and transcriptome of healthy subjects and metabolic syndrome (MetS) patients [25]. Subjects were orally exposed to a single dose of either rich- or low-polyphenols EVOO and after 4 h gene expression microarrays were carried out on peripheral blood mononuclear cells (PBMCs). In healthy subjects, rich-polyphenols EVOO induced evident changes in many mRNA expression coding for genes involved in inflammation (see below) and cancer (upregulation of the DNA Damage Response systems, suppression of different pathways involved in cell and cancer proliferation such as ERK/MAPK, CXCR4, HGF/EGF, HIF1α signaling cascades). Some of the genes involved in the cancer upset were confirmed by RTqPCR including retinoid X receptor beta, heat shock 70 kDa protein 1A, cyclin K and others [25]. At the same time, rich-polyphenols EVOO intake reduced the expression of two oncogenic miRNAs (miRNA-19a-3p and miR-181b-5p) and upregulate the expression of a tumour suppressor miRNA (miR-23b-3p). Very importantly, most of these changes were not observed after low-polyphenol EVOO challenge and were less evident in MetS patients [25]. Although very interesting, this study showed essentially an association between miRNA abundance, genes expression and anti-cancer activities. Further studies are necessary to demonstrate the causal relationships between these effects.

#### 2.1.2. Anti-Inflammatory Effects

As above reported, in addition to the anti-cancer properties two studies, one on rats [61] and one on humans [25] showed that EVOO was able to modulate miRNAs and genes with anti-inflammatory properties. In DMH-treated rats, EVOO intake downregulated the mRNA expression of the transcription factor NF-κB and its target genes VEGF and MMP-9 [61]. In humans, rich-polyphenols EVOO intake suppressed the expression of interleukin-1 receptor-associated kinase 3, which is involved in the regulation in the NF-κB and IL-8 signaling, and upregulated the anti-inflammatory miRNA-23b-3p miRNAs [25].

The anti-inflammatory effect of hydroxytyrosol and oleuropein was investigated in vitro in a cell line of murine macrophages RAW264.7 and in human granulocytes/monocytes [28]. At low, nutritionally relevant concentration (10 µM), these compounds inhibited the PMA-induced activation of granulocytes and monocytes. In murine macrophages, hydroxytyrosol and oleuropein reduced the LPS induced nitrites and PGE production, while repressed the LPS induced up regulation of miRNA-146a and induced nuclear translocation of NRf2 ((erythroid-derived 2)-like 2) [28]. Generally, hydroxytyrosol was more active than oleuropein. In another in vitro system, hydroxytyrosol was able to counteract adipocytes inflammation induced by TNF-α [35]. Among other effects, hydroxytyrosol prevented the TNF-induced ROS production, NF-κB activation and upregulation of MCP-1, CXCL-10, M-CSF, IL-1, VEGF, COX-2 and MMP-2 both at mRNA and protein levels in Simpson–Golabi–Behmel Syndrome adipocytes [35]. In parallel, hydroxytyrosol prevented the TNF-induced upregulation of miRNA-34a and miRNA-155 levels as well as the downregulation of let-7c levels in both cells and exosomes. Essentially, similar effects in the same experimental system of human adipocytes have also been shown for oleocanthal and oleacein [37]. Furthermore, an upregulation of let-7 miRNA was observed in human umbilical and retinal endothelial cells stimulated with IL-1β after treatment with hydroxytyrosol-3-O-sulphate, the major hydroxytyrosol plasma metabolite [38]. In this cellular system, hydroxytyrosol-3-O-sulphate also prevented IL-1β induced endothelial-to-mesenchymal transition [38].

#### 2.1.3. Other Effects

The ability of olive oil phenolic compounds to modulate the expression of miRNA in relation to other biological activities has been investigated (Table S1). A possible anti-osteoarthritis effect of hydroxytyrosol mediated by modulation miRNA was reported on both human primary and C-28/I2 chondrocytes [27]. This phenol prevented the upregulation of miRNA-9 and the downregulation of SIRT-1 induced by treatment of chondrocytes with H_2_O_2_. Very importantly, the causal relationship between the upregulation of miRNA-9 expression and the downregulation of SIRT-1 was clearly demonstrated by both silencing experiments and luciferase-based gene reporter assay [27]. These experiments allowed to conclude that the protective action of hydroxytyrosol against the damaging effects of oxidative stress in chondrocytes and osteoarthritis-related effects was mediated by miRNA-9 [27]. The same authors have further demonstrated that the effects of hydroxytyrosol (and oxidative stress) on miRNA-9 expression were mediated by interfering with the promoter methylation [42]. Essentially, these treatments modulated miRNA-9 expression by exerting opposite effects on the promoter methylation status, with oxidative stress reducing and HT rescuing and sustaining the hypermethylation of CpG islands [42]. Although very interesting, these data obtained with pharmacological doses of hydroxytyrosol (100 µM) make it difficult to predict whether the daily intake of this compound with a diet may have any physiological relevance in the prevention of osteoarthritis. An anti-oxidant effect, in terms of reduction of both ROS and malondialdehyde production, has been reported for the hydroxytyrosol-oleate at more physiological concentration (5 µM) on human keratinocytes [32]. These effects were associated with the upregulation of miRNA-34a, miRNA-21 and miRNA-29a [32]. Whether a causal relationship exists between these two phenomena remains to be determined.

In an aging mouse model, it was found that EVOO rich in phenols (H-EVOO: 718.8 mg/kg) was able to counteract the age-related decline in motor coordination, spatial memory and anxiety-related behaviour [29]. These behavioural modifications were compared with mice fed a diet containing low phenols EVOO (L-EVOO: 9.3 mg/kg) and were associated with changes in gene and microRNA expression in brain. In particular, H-EVOO was able to counteract the downregulation of some brain genes that occurs during aging. Among them, a significant upregulation of genes associated with synaptic plasticity and with motor and cognitive behaviour including Notch1, BMPs (bone morphogenetic proteins), NGFR (nerve growth factor receptor), GLP1R (glucagon-like peptide-1receptor) and CRTC3 (CREB-regulated transcription coactivator 3) was observed [29]. At the same time, H-EVOO downregulated sixty-three miRNAs, out of 1203 analysed, resulting in a mice cortex miRNA expression profiles similar to those observed in young mice [29].

Another study on rats has shown that maternal consumption of different types of fatty acids during early pregnancy influences miRNA expression in both maternal and offspring tissues [23]. In particular, olive oil used as a source of n-9 fatty acid, upregulated a series of miRNA in offspring tissues compared to the soybean oil, including miR-215, miR-10b, miR-26, miR-377-3p, miR-21, and miR-192. However, the presence and concentration of phenols in olive oil were not mentioned. In addition, similar effects were also obtained with other oil such as fish oil, linseed oil and palm oil leading to excluding a role of phenolic compounds [23].

The possible beneficial effect of hydroxytyrosol on cognitive impulsivity and anxiety, an attribute common to different neurodegenerative diseases including Alzheimer’s disease (AD), was investigated in a mouse model of oA42i- (soluble oligomeric amyloid β1−42 plus ibotenic acid) induced AD [39]. Hydroxytyrosol prevented several deleterious effects induced by oA42i. It improved the impulsive decision-making and attenuated the anxiety-like behaviour in the oA42i-challenged mice. This effect was associated with an upregulation in the mice hippocampi of miRNA-124, which was decreased by oA42i treatment, and to a downregulation of HDAC6 and HSP90 proteins, which were increased by oA42i treatment [39]. Interestingly, hydroxytyrosol induced a transcriptional activation of CRTC1, an effect similar to that observed for the CRTC3 in the aging-model reported above [29]. The in vivo findings were confirmed in vitro on organotypic hippocampal slice cultures (OHSCs) using a dose of hydroxytyrosol of 100 µM. Since the route of administration and the dose of hydroxytyrosol used in the in vivo experiments have not been reported, no preventive role on AD in humans can be defined under conditions of normal dietary intake of this compound.

**Figure 2:**
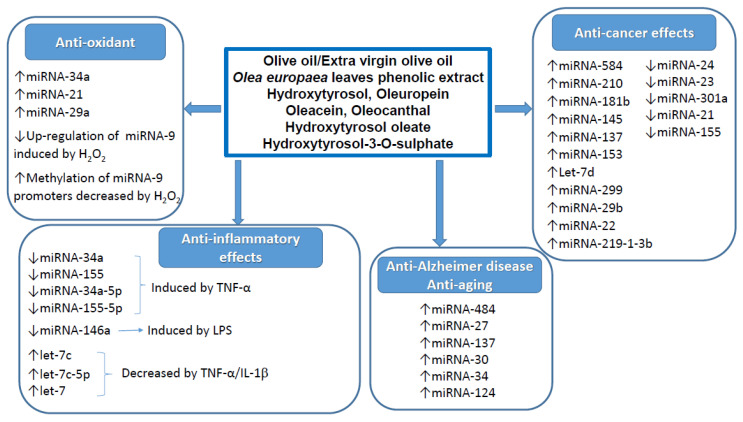
Main effects of olive oil and/or its phenolic compounds on miRNAs expression in relation to different biological and healthy properties. “↑” and “↓” indicate increment and decrement of miRNA expression, respectively.

Two further studies have suggested that the miRNA modulating properties of hydroxytyrosol could be, at least in part, involved in the regulation of genes correlated to the oxidative stress, lipid metabolism and other metabolic processes [24,33]. Several miRNAs were modulated in different organs of mice fed with hydroxytyrosol. Administration of this compound increased triglyceride levels [24]. In particular, one-week supplementation with hydroxytyrosol resulted in an increased expression of miRNA-193a-5p in human healthy subjects [24]. In a further transcriptomic analysis of mice, two novel potential hydroxytyrosol target genes were found, i.e., Fgf21 and Rora [33]. Once again, these studies suggest, but do not prove, that hydroxytyrosol-modulated miRNAs contribute to the regulation of genes involved in oxidative stress, lipid metabolism and other metabolic processes. The most relevant effects exerted by olive oil and its phenols on miRNAs expression in relation to the different biological and healthy properties are shown in Figure 2.

### 2.2. DNA Methylation

The characteristics of the 15 selected studies investigating the effects of olive oil and its phenolic compounds on DNA methylation are shown in Table S2. Five studies reported data in vitro on cell systems [46,53,59,60,63], 8 studies showed results on animal models [40,41,42,43,48,58,60,61] and 3 studies reported results on human trials [39,45,47]. Eleven studies were carried out with olive oil [39,40,41,42,43,45,47,58,59,60,61], 2 articles reported data on oleacein [46,63], one study each investigated the effect of hydroxytyrosol [48] and of an extract obtained from *Olea europaea* leaves [44].

#### 2.2.1. Anti-Cancer Effects

Three studies have investigated the chemopreventive potential of EVOO on the rat model of carcinogenesis, two of them using DMBA to induce breast cancer [41,58] and one using DMH to induce colon cancer [61]. Surprisingly, the data on breasts did not show any cancer preventive activity by EVOO [41] in one case or even a deleterious effect in the other [58]. In contrast, EVOO was very effective in reducing incidence, multiplicity and size of colon tumours [61]. Several effects on DNA methylation were observed in both animal models. In particular, discordant results were obtained regarding the mRNA expression of DNMT3a in breast cancer cells, which was downregulated in one study [58] and upregulated in the other [41]. Instead, the preventive effect on colon cancer was associated with: (i) an increase of methylation of promoter region of NF-κB, VEGF, MMP-9 coupled to a downregulation of relative genes expression; (ii) a decrease of methylation of promoter region of miR-143, miR-145, caspase-3 and caspase-9 coupled to an upregulation of caspase-3 and caspase-9 genes expression [61]. The main limit of these studies is that they focalized the attention on fats (olive oil was used as n-9 monounsaturated fatty acid source) and did not consider the presence or not of phenolic compounds in EVOO. The phenolic profile of the EVOO used in these studies may deeply influence its epigenetic effects. Indeed, a study on human colon cells demonstrated that the ability of phenol rich EVOO to upregulate the promoter methylation of CNR1 gene and downregulate its expression was lost after removing phenols (rectified olive oil: ROO) [60]. In addition, oleacein, one of the most abundant phenols present in EVOO, was able to inhibit tumorigenesis both in vitro and in vivo (xenograft transplantation) of breast cancer steam cells [46]. Oleacein, acting as SAM competitive inhibitor, was able to inhibit DNMT1, 3A/3L and 3B/3L enzyme activity in breast cancer cells nuclear extract [46]. An anti-cancer activity of oleacein was also reported against multiple myeloma cells, although no effect of global DNA methylation and mRNA and protein expression of DNMT1, DNMT3A and DNMT3B was observed [63]. In contrast to oleacein, hydroxytyrosol was able to increase the global DNA methylation of pig fetus after supplementation of the maternal diet [48]. This effect was associated with an improved fetal antioxidant status and glucose metabolism. Finally, treatment of human primary GBM tumour cells with an *Olea europaea* leaf extract inhibited the proliferation, decreased p53 expression and caused an increment of O6-methylguanine-DNA-methyltransferase (MGMT) promoter methylation [44]. All of this evidence suggests that the chemopreventive activities of EVOO phenols may be correlated to its ability to modulate the methylation status of important genes involved in the carcinogenesis process.

#### 2.2.2. Anti-Inflammatory Effects

As reported above, EVOO exerted anti-inflammatory activity in the colon of DMH treated rat favoring the hypermethylation of promoter region of NF-κB and silencing its mRNA and protein expression [61]. Similarly, Sprague–Dawley rats fed a diet containing 10% fat derived from different oils (coconut, sunflower and olive) showed both an increment of TNFα promoter methylation and a decrease of TNFα both mRNA expression and release in adipocytes of olive oil fed animals compared to coconut oil [42]. In the same experimental system, it was also found that olive oil reduced the VEGFB (vascular endothelium grown factor b) promoter methylation levels in rat visceral and subcutaneous adipose tissue and increased the VEGFB gene and protein expressions [43]. However, these effects were small and no evidence was provided about their possible real functional consequences on obesity, inflammation and other associated metabolic diseases. Further evidence on anti-inflammatory properties of EVOO was provided by an in vitro study showing a preventive activity toward lipopolysaccharides (LPS)-induced inflammation on human macrophages (THP-1 cells) [59]. EVOO was particularly efficient in restoring a normal level of some inflammatory genes such as IL-6, IL-1β and MCP-1. At the same time, EVOO caused a reduction of both DNMT3A and DNMT3B mRNA expression. In addition, it prevented the increase of TET2 expression and the reduction of global DNA methylation induced by inflammation in macrophages [59]. Unfortunately, these in vitro results on IL-6 were not reproduced in a human intervention trial with EVOO on cyclists in which a pro-inflammatory state was induced by acute aerobic exercise [47]. In this case, although EVOO significantly reduced the IL6 CpG3 DNA methylation in comparison with n-3 PUFA supplementation, no significant effect was observed regarding the IL-6 mRNA expression [47]. Furthermore, no significant effects by EVOO were also observed on global DNA methylation and mRNA expression of DNMT3a and DNMT3b, while a reduction of mRNA expression of DNMT1 was evident [47]. It is important to underline that all these studies that have tried to correlate the anti-inflammatory activities with the modulation of DNA methylation status were carried out with EVOO for which the phenolic composition was not reported. This makes it impossible to hypothesize the involvement of phenols in these effects. Further studies comparing the effect of high- or low- phenol EVOO will shed some light on this question.

#### 2.2.3. Other Effects

**Figure 3:**
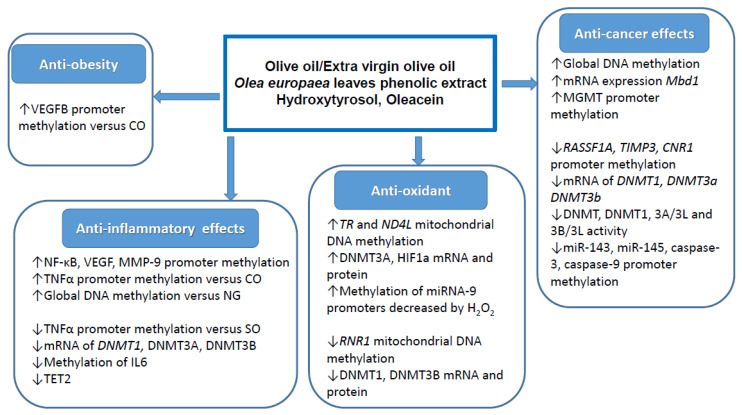
Main effects of olive oil and/or its phenolic compounds on DNA methylation in relation to different biological and healthy properties. “↑” and “↓” indicate increment and decrement of DNA methylation, respectively.

Two additional human trials were performed with olive oil [39] or EVOO within a Mediterranean diet [45] to reveal their potential effect on DNA methylation. In the first case, the effects of olive oil on DNA methylation of genes involved in PUFA biosynthesis in peripheral blood mononuclear cells of chronic kidney disease patients were compared with n-3 LC-PUFA. It was found that the olive oil induced methylation increased of CpGs in some 5′ regulatory regions of specific genes was associated with a repression of FADS2, FADS1, ELOVL5 (only female) and ELOVL2 mRNA expression [39]. These data are important for understanding the effect of fatty acids on PUFA metabolism and cell function. The other human intervention trial was aimed to study the influence of two Mediterranean diets, one rich in EVOO and the other one in nuts on the methylation status of peripheral white blood cell genes [45]. A low-fat diet was used as control. The results indicated that, after five years of intervention, the EVOO rich Mediterranean diet prevented diabetes, hypercholesterolemia and arterial hypertension. Among the 223 CpGs screened, a decrease in DNA methylation of cg17071192–GNAS/GNASAS was found in subjects following the EVOO rich Mediterranean diet compared to both nut rich diet and control diet [45]. GNASAS encodes for an antisense RNA transcript that regulates GNAS, which has been involved in glucose and energy regulation. Although these findings may have implications for understanding the epigenetic mechanisms behind the health effects of olive oil, no direct evidence was provided for their real physiological consequences. In addition, once again, the role of phenols on these effects was not considered. The most relevant effects exerted by olive oil and its phenols on DNA methylation in relation to the different biological and healthy properties are shown in Figure 3.

### 2.3. Histone Modification

The characteristics of the 13 selected studies investigating the effects of olive oil and its phenolic compounds on histone modification are shown in Table S3. Eight studies reported data in vitro on different cell systems [49,53,54,55,56,57,59,63], 3 studies showed results on animal models [50,58,62] and 2 studies reported results ex vivo on freshly isolated cells from human tissue [51,52]. Two studies investigated the effects of hydroxytyrosol [51,62], 3 articles reported data on oleuropein [50,54,57], 3 studies were carried out with olive oil [52,58,59], and one study with phenolic extract obtained from olive oil [49]. Four investigations reported data on oleacein [53,55,56,63].

#### 2.3.1. Anti-Cancer Effects

In addition to the epigenetic effects above reported, the histone modifications have also been considered hallmarks of cancer [64]. These reversible epigenetic effects may be induced by nutritional factors, including EVOO and its phenolic compounds. Indeed, out of 13 selected studies, 8 articles have correlated the histone modifications with an anti-cancer activity of the phenols [49,53,54,55,56,57,58,63]. Two of these articles were already discussed above [58,63]. The first was the only study to investigate the ability of EVOO to modify histone proteins on an animal model. In the DMBA induced breast cancer in rats, EVOO reduced the H4K16ac levels in mammary gland and in tumour tissue while the H4K20me3 was decreased only in tumour tissue [58]. Instead, H3K27me3 and H3K4me2 were not significantly affected [58]. Since lysine acetylation and methylation of histones, such as H4K16ac, H4K20me3, H3K27me3 and H3K4me2, have been frequently associated with breast cancer, these results support the possible preventive effect of EVOO on this disease. In the second study, the anti-cancer effects of oleacein on several myeloma multiple cell lines were associated with a reduced expression levels of both mRNA and protein of class I/II histone deacetylases, and with an increased acetylated histones H3 and H4 [63]. These effects were not due to an inhibition of HDAC activity but were associated with a downregulation of Sp1, the major Caspase 8 dependent trans-activator of HDACs promoter. Interestingly, oleacein synergistically enhanced the anti-cancer activity of the clinically relevant proteasome inhibitor carfilzomib. This combination reduced the viability and increased apoptosis in NCI-H929-treated cells [63]. Further in vitro studies demonstrated that oleacein was able to suppress the overproduction of oncometabolite R-2-hydroxyglutaratye (2HG) in both MCF10A and HCT116 (both wild type and heterozygous IDH1-R132H mutation expressing cells) by inhibiting the enzymatic activity of the recombinant mutant isocitrate dehydrogenase 1 (R132HIDH1) [53]. These effects were associated with a reduction of both the H3K9me3 levels and the number and size of colonies of MCF10A and HCT116 cells [53]. Cuyas et al. further confirmed the epigenetics-mediated anticancer effects of oleacein [55,56]. By using a systematic chemoinformatics approach coupled to laboratory-based confirmatory testing, the authors showed that, in a low-micromolar physiological concentration range (<20 μmol/L), oleacein was able to inhibit the activity of lysine-specific demethylase 6A and *N*-methyltransferase 4, together with the suppression of acetyl-CoA-synthesizing enzyme (ACLY) and nicotinamide *N*-methyltransferase (NNMT), two metabolic enzymes associated with many types of tumours [55,56]. Moreover, oleacein was able to suppress the demethylase activity of LSD1 (KDM1A) (IC50 of ~2.5 µmol/L). This phenomenon can be explained in terms of the ability of oleacein to inhibit transcriptional factor SOX2 in breast cancer stem cells, at protein and mRNA level, via LSD1 inhibition [55,56].

In MCF7 breast cancer cells, oleuropein exerted anti-cancer effects by reducing the cell viability and migration, and inducing apoptosis. These effects were associated with a downregulation of HDAC2 and HDAC3 expression, two genes highly expressed in the more aggressive subgroups of breast cancer [54]. In the same cellular model, oleuropein was also able to reduce the mRNA expression of HDAC4. Similarly, this epigenetic effect was associated with a cytotoxicity on MCF7 cells, and to a pro-apoptotic and anti-migration ability [57]. Although further studies are need, these data suggest that oleuropein can be a safe cancer preventive and therapeutic agent acting on epigenetic mechanisms including HDAC inhibition.

In addition to the single compounds, the epigenetic effects of complex phenolic mixtures isolated from EVOO were also studied. In particular, Oliveras-Ferraros et al. investigated in vitro epigenetic effects of a crude phenolic extracts (EVOO-PE) obtained from 14 monovarietals of Spanish EVOO on JIMT-1 breast cancer cell line [49]. They observed that EVOO-PE treatment incremented the H3K18ac levels. The anti-cancer effects of crude extracts were associated with the inhibition of cell growth and the arrest at the G2/M phase of the cell cycle. These activities were positively correlated with total phenolic indexes and secoiridoids content, while they were inversely correlated with the content of lignans [49]. The EVOO-PE induced effects on the JIMT-1 transcriptome and the consequent key pathways/functions potentially associated with the degree of anti-tumour activity were further analysed. It was found that the enriched gene set responsible for the differential efficacy of secoiridoids-rich versus secoiridoids-low/null EVOO-PE was the cell cycle and p53 signaling pathway, in particular CREBBP, CDKN1A (p21, Cip1), CDKN1C (p57, Kip2) and PMAIP-1 (Noxa, APR) and GADD45 genes. Moreover, the ability of EVOO-PE to inhibit JIMT-1 cell growth and to activate GADD45 genes was closely related to their ability to activate MEK1, p38 MAPK, Stat3, and NF-κB p65. As reported above, these effects were correlated with the secoiridoids content in the EVOO-PE [49].

#### 2.3.2. Anti-Inflammatory Effects

Out of the 13 selected studies, just one study investigated the EVOO induced histone modifications in association with the anti-inflammatory activity [59]. In addition to the epigenetics effects described above regarding the miRNA and DNA methylation, EVOO was able to restore the overexpression levels of HDAC1 and HDAC3 induced by low-level inflammation in the THP-1 cell line. This effect was associated with a restoration of membrane fluidity, which resulted in being altered by inflammatory stimuli [34]. Various studies showed the ability of olive oil polyphenols, such as oleuropein and oleacein, to decrease the HDACs, but these results have been discussed in terms of anti-cancer effects in the previous section.

#### 2.3.3. Other Effects

Recently, histone acetylation is emerging as an important element in the pathogenesis of neurologic disorders such as Alzheimer’s disease (AD) [65]. Histone acetylation ameliorated cognitive deficits in AD animal models, and its targeting is considered a novel promising therapeutic strategy to treat AD [66]. Based on the experimental and epidemiological evidence of health benefits on neurodegenerative diseases via epigenetic actions exerted by many plant polyphenols [67], olive oil polyphenols have been investigated for their potential histone acetylation activity on AD [50]. In this regard, hydroxytyrosol and oleuropein were studied. As previously mentioned, it was found that hydroxytyrosol in vivo, in a mouse model of oA42i (soluble oligomeric amyloid β1−42plus ibotenic acid)-induced AD, increased miRNA124, which was decreased by oA42i treatment. This effect seems to be correlated with the decrease of HDAC6, increased by oA42i treatment, assuming that HDAC is the downstream target of miRNA-124 [62]. Oleuropein increased the levels of H3K9ac and H4K5ac in the brain of TgCRND8 mice; moreover, it decreased the HDAC2 levels [50]. In the same AD mouse model, oleuropein reduced functional deficit, decreased the glutamynil cyclase expression and induced autophagy. The anti-AD effect of oleuropein was also demonstrated by a reduction of pE3-Aß and Aß42 plaques in the cortex and hippocampus of AD mouse model and in vitro modifying the aggregation path of pE3-Aß and skipping or reducing the presence of toxic intermediates [50].

One ex vivo study investigated the histone modification induced by hydroxytyrosol in correlation with diabetes, in particular on wound formation and healing [51]. This study was carried out in tissue sections obtained from the peri wound of a diabetic ulcer treated with a commercial topical product, containing the Olivamine 10^®^ formulation, which includes a hydroxytyrosol containing olive extract. It was found an increment of HDAC6 level, a α-tubulin deacetylase, without any quantitative change in the acetylated α-tubulin levels, suggesting the ability to promote chemotactic cell movement [51]. Moreover, it was demonstrated in vitro the ability of hydroxytyrosol to inhibit the LSD1 demethylase (IC50 of 3.57 µM), a key histone modifier involved in the maintenance of gene expression in human embryonic stem cells [68]. LSD1 demethylase is overexpressed in many cancer types [69], and its inhibition has been shown to mitigate cellular proliferation and invasion of neoplastic cells. Certainly, more investigations are needed to know both the effect of HDAC6 expression on wound healing and, in particular, whether the expressional increase in HDAC6 within the tissue was due to hydroxytyrosol specifically or to the synergistic effect of the Olivamine 10^®^ compounds [51].

**Figure 4:**
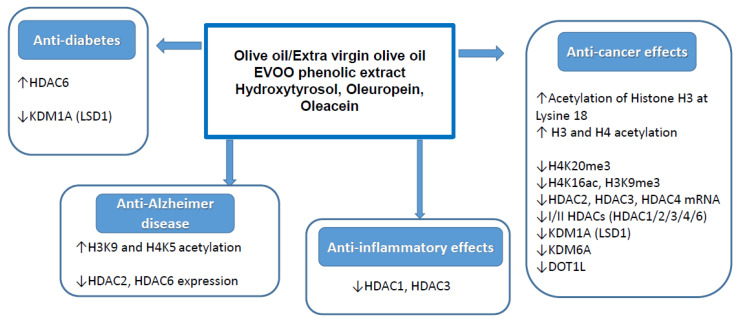
Main effects of olive oil and/or its phenolic compounds on histone modification in relation to different biological and healthy properties. “↑” and “↓” indicate increment and decrement of histone modification, respectively.

To support the evidence that maternal diet modifies epigenetic programming in offspring, with consequences on the regulation of the immune system, an interesting study investigated the role of maternal intake of EVOO on placental histone acetylation in immune regulatory genes [52]. Authors found that H3 acetylation levels at the promoters of FOXP3, interleukin 10 receptor subunit alpha (IL10RA) and interleukin 7 receptor (IL7R) genes were significantly increased in the placenta specimens from antroposophic mothers who regularly used EVOO respect non-antroposophic mothers [52]. These data suggest that compounds in olive oil may have particularly important effects on the histone marks in placenta. These effects were not associated with demographic variables that could influence placenta acetylation (maternal age at birth, parity, birth weight gestational age, or parental smoking). Although this study did not investigate the way in which H3 and H4 acetylation differences are reflected at the gene expression level, the authors speculate that acetylation may lead to elevated transcriptional activities of these genes that could promote the accessibility of FOXP3, IL10RA and IL7R promoters to the transcriptional machinery. This may affect early innate immune responses, anti-inflammatory mechanisms [70,71], generation of regulatory T-cells and tolerance [72]. As previously discussed, some polyphenols present in EVOO showed the H3 acetylation ability by downregulating HDAC2, HDAC6 and the lysine-specific histone demethylase 1 (LSD1) such as oleuropein [50] and hydroxytyrosol, respectively [51,52]. The most relevant effects exerted by olive oil and its phenols on histone modification in relation to the different biological and healthy properties are shown in Figure 4.

## 3. Methods

In this study, the PRISMA (Preferred Reporting Items for Systematic Reviews and Meta-Analyses) statements were followed [73]. In order to find out articles investigating the epigenetic effects of EVOO and olive oil secoiridoid phenols, we carried out a systematic electronic literature search in April 2020, without restrictions, on PubMed (http://www.ncbi.nlm.nih.gov/pubmed/), Web of Science (http://wokinfo.com/) and Scopus (https://www.scopus.com/home.uri) databases. The following key words were used: (“olive oil” OR hydroxytyrosol OR oleuropein OR oleocanthal OR oleacein OR secoiridoids) AND (“DNA methylation” OR “histone modifications” OR miRNA OR epigenetic). In addition, the reference lists of included articles and recent significant reviews were manually examined to identify additional relevant publications. Potential identified articles were included if they reported data on the epigenetic effects of olive oil phenols on different models both in vivo and in vitro. Only data from experimental studies were considered, the “in silico” and “bioinformatics” data were not considered. The epigenetic modifications considered were: DNA methylation, post-translational histone modifications (acetylation and methylation) and miRNA expression. These effects may be correlated to each other; for instance, the expression of miRNAs may be regulated by other epigenetic mechanisms such as DNA methylation and histone modifications. Indeed, some studies investigated more than one single epigenetic modification. From the selected studies, we extracted the following information: first author’s last name, year of publication, compound tested, study model adopted, epigenetic effect and other relevant effects.

## 4. Conclusions

Several studies have investigated the possible correlation between the biological activities of olive oil and its phenolic compounds with epigenetic modifications. The findings summarized in the present systematic review may help to understand the role played by epigenetic mechanisms behind the health properties of olive oil. The effects of phenols on miRNAs expression have received the majority of attention. However, as the studies were conducted in very different ways in terms of cell systems, compounds tested and miRNAs measured, it is difficult to suggest a single shared effect. It is remarkable to note that most of the studies on DNA methylation were done with EVOO, the phenolic composition of which was not reported. In addition, no direct evidence was provided for the causal relationships between epigenetics modification and health related effects. Therefore, further studies are necessary to demonstrate the real physiological consequences of the epigenetics modification induced by EVOO and its phenolic compounds.

## Data Availability

Not applicable.

## Supplementary Materials

Supplementary materials are available online: Tables S1–S3.

## References

[1] Mazzocchi A., Leone L., Agostoni C., Pali-Schöll I. (2019). The Secrets of the Mediterranean Diet. Does [Only] Olive Oil Matter?. Nutrients.

[2] Galbete C., Schwingshackl L., Schwedhelm C., Boeing H., Schulze M.B. (2018). Evaluating Mediterranean diet and risk of chronic disease in cohort studies: An umbrella review of meta-analyses. Eur. J. Epidemiol..

[3] Soltani S., Jayedi A., Shab-Bidar S., Becerra-Tomás N., Salas-Salvadó J. (2019). Adherence to the Mediterranean diet in relation to all-cause mortality: A systematic review and dose-response meta-analysis of prospective cohort studies. Adv. Nutr..

[4] Foscolou A., Critselis E., Panagiotakos D. (2018). Olive oil consumption and human health: A narrative review. Maturitas.

[5] De Santis S., Cariello M., Piccinin E., Sabbà C., Moschetta A. (2019). Extra virgin olive oil: Lesson from nutrigenomics. Nutrients.

[6] Gaforio J.J., Visioli F., Alarcón-de-la-Lastra C., Castañer O., Delgado-Rodríguez M., Fitó M., Hernández A.F., Huertas J.R., Martínez-González M.A., Menendez J.A. (2019). Virgin olive oil and health: Summary of the III international conference on virgin olive oil and health consensus report, JAEN (Spain) 2018. Nutrients.

[7] Jimenez-Lopez C., Carpena M., Lourenço-Lopes C., Gallardo-Gomez M., Lorenzo J.M., Barba F.J., Prieto M.A., Simal-Gandara J. (2020). Bioactive compounds and quality of extra virgin olive oil. Foods.

[8] Servili M., Esposto S., Fabiani R., Urbani S., Taticchi A., Mariucci F., Selvaggini R., Montedoro G.F. (2009). Phenolic compounds in olive oil: Antioxidant, health and organoleptic activities according to their chemical structure. Inflammopharmacology.

[9] Gorzynik-Debicka M., Przychodzen P., Cappello F., Kuban-Jankowska A., Marino Gammazza A., Knap N., Wozniak M., Gorska-Ponikowska M. (2018). Potential health benefits of olive oil and plant polyphenols. Int. J. Mol. Sci..

[10] Marković A.K., Torić J., Barbarić M., Brala C.J. (2019). Hydroxytyrosol, tyrosol and derivatives and their potential effects on human health. Molecules.

[11] Fabiani R. (2016). Anti-cancer properties of olive oil secoiridoid phenols: A systematic review of in vivo studies. Food Funct..

[12] George E.S., Marshall S., Mayr H.L., Trakman G.L., Tatucu-Babet O.A., Lassemillante A.M., Bramley A., Reddy A.J., Forsyth A., Tierney A.C. (2019). The effect of high-polyphenol extra virgin olive oil on cardiovascular risk factors: A systematic review and meta-analysis. Crit. Rev. Food Sci. Nutr..

[13] Torić J., Marković A.K., Brala C.J., Barbarić M. (2019). Anticancer effects of olive oil polyphenols and their combinations with anticancer drugs. Acta Pharm..

[14] Serreli G., Deiana M. (2020). Extra virgin olive oil polyphenols: Modulation of cellular pathways related to oxidant species and inflammation in aging. Cells.

[15] Casamenti F., Stefani M. (2017). Olive polyphenols: New promising agents to combat aging-associated neurodegeneration. Expert Rev. Neurother..

[16] Piroddi M., Albini A., Fabiani R., Giovannelli L., Luceri C., Natella F., Rosignoli P., Rossi T., Taticchi A., Servili M. (2017). Nutrigenomics of extra-virgin olive oil: A review. Biofactors.

[17] Anderson O.S., Sant K.E., Dolinoy D.C. (2012). Nutrition and epigenetics: An interplay of dietary methyl donors, one-carbon metabolism and DNA methylation. J. Nutr. Biochem..

[18] Bishop K.S., Ferguson L.R. (2015). The interaction between epigenetics, nutrition and the development of cancer. Nutrients.

[19] Esquela-Kerscher A., Slack F.J. (2006). Oncomirs—microRNAs with a role in cancer. Nat. Rev. Cancer.

[20] Jiménez-Chillarón J.C., Díaz R., Martínez D., Pentinat T., Ramón-Krauel M., Ribó S., Plösch T. (2012). The role of nutrition on epigenetic modifications and their implications on health. Biochimie.

[21] Tunca B., Tezcan G., Cecener G., Egeli U., Ak S., Malyer H., Tumen G., Bilir A. (2012). Olea europaea leaf extract alters microRNA expression in human glioblastoma cells. J. Cancer Res. Clin. Oncol..

[22] Tezcan G., Tunca B., Bekar A., Budak F., Sahin S., Cecener G., Egeli U., Taskapilioglu M.O., Kocaeli H., Tolunay S. (2014). Olea europaea leaf extract improves the treatment response of GBM stem cells by modulating miRNA Expression. Am. J. Cancer Res..

[23] Casas-Agustench P., Fernandes F.S., Tavares Do Carmo M.G., Visioli F., Herrera E., Dávalos A. (2015). Consumption of distinct dietary lipids during early pregnancy differentially modulates the expression of microRNAs in mothers and offspring. PLoS ONE.

[24] Tomé-Carneiro J., Crespo M.C., Iglesias-Gutierrez E., Martín R., Gil-Zamorano J., Tomas-Zapico C., Burgos-Ramos E., Correa C., Gómez-Coronado D., Lasunción M.A. (2016). Hydroxytyrosol supplementation modulates the expression of miRNAs in rodents and in humans. J. Nutr. Biochem..

[25] D’Amore S., Vacca M., Cariello M., Graziano G., D’Orazio A., Salvia R., Sasso R.C., Sabbà C., Palasciano G., Moschetta A. (2016). Genes and miRNA expression signatures in peripheral blood mononuclear cells in healthy subjects and patients with metabolic syndrome after acute intake of extra virgin olive oil. Biochim. Biophys. Acta.

[26] Xu T., Xiao D. (2017). Oleuropein enhances radiation sensitivity of nasopharyngeal carcinoma by downregulating PDRG1 through HIF1α-repressed microRNA-519d. J. Exp. Clin. Cancer Res..

[27] D’Adamo S., Cetrullo S., Guidotti S., Borzì R.M., Flamigni F. (2017). Hydroxytyrosol modulates the levels of microRNA-9 and its target sirtuin-1 thereby counteracting oxidative stress-induced chondrocyte death. Osteoarthr. Cartil..

[28] Bigagli E., Cinci L., Paccosi S., Parenti A., D’Ambrosio M., Luceri C. (2017). Nutritionally relevant concentrations of resveratrol and hydroxytyrosol mitigate oxidative burst of human granulocytes and monocytes and the production of pro-inflammatory mediators in LPS-stimulated RAW 264.7 macrophages. Int. Immunopharmacol..

[29] Luceri C., Bigagli E., Pitozzi V., Giovannelli L. (2017). A nutrigenomics approach for the study of anti-aging interventions: Olive oil phenols and the modulation of gene and microRNA expression profiles in mouse brain. Eur. J. Nutr..

[30] Xing Y., Cui D., Wang S., Wang P., Xing X., Li H. (2017). Oleuropein represses the radiation resistance of ovarian cancer by inhibiting hypoxia and microRNA-299-targetted heparanase expression. Food Funct..

[31] Abtin M., Alivand M.R., Khaniani M.S., Bastami M., Zaeifizadeh M., Derakhshan S.M. (2018). Simultaneous downregulation of miR-21 and miR-155 through oleuropein for breast cancer prevention and therapy. J. Cell. Biochem..

[32] Benincasa C., La Torre C., Plastina P., Fazio A., Perri E., Caroleo M.C., Gallelli L., Cannataro R., Cione E. (2019). Hydroxytyrosyl oleate: Improved extraction procedure from olive oil and by-products, and in vitro antioxidant and skin regenerative properties. Antioxidants.

[33] López de Las Hazas M.C., Martin-Hernández R., Crespo M.C., Tomé-Carneiro J., Del Pozo-Acebo L., Ruiz-Roso M.B., Escola-Gil J.C., Osada J., Portillo M.P., Martinez J.A. (2019). Identification and validation of common molecular targets of hydroxytyrosol. Food Funct..

[34] Tezcan G., Aksoy S.A., Tunca B., Bekar A., Mutlu M., Cecener G., Egeli U., Kocaeli H., Demirci H., Taskapilioglu M.O. (2019). Oleuropein modulates glioblastoma miRNA pattern different from olea europaea leaf extract. Hum. Exp. Toxicol..

[35] Scoditti E., Carpi S., Massaro M., Pellegrino M., Polini B., Carluccio M.A., Wabitsch M., Verri T., Nieri P., De Caterina R. (2019). Hydroxytyrosol modulates adipocyte gene and miRNA expression under inflammatory condition. Nutrients.

[36] D’Adamo S., Cetrullo S., Borzì R.M., Flamigni F. (2019). Effect of oxidative stress and 3-Hydroxytyrosol on DNA methylation levels of miR-9 promoters. J. Cell. Mol. Med..

[37] Carpi S., Scoditti E., Massaro M., Polini B., Manera C., Digiacomo M., Esposito Salsano J., Poli G., Tuccinardi T., Doccini S. (2019). The extra-virgin olive oil polyphenols oleocanthal and oleacein counteract inflammation-related gene and miRNA expression in adipocytes by attenuating NF-ΚB activation. Nutrients.

[38] Terzuoli E., Nannelli G., Giachetti A., Morbidelli L., Ziche M., Donnini S. (2020). Targeting endothelial-to-mesenchymal transition: The protective role of hydroxytyrosol sulfate metabolite. Eur. J. Nutr..

[39] Hoile S.P., Clarke-Harris R., Huang R.C., Calder P.C., Mori T.A., Beilin L.J., Lillycrop K.A., Burdge G.C. (2014). Supplementation with n-3 long-chain polyunsaturated fatty acids or olive oil in men and women with renal disease induces differential changes in the DNA methylation of FADS2 and ELOVL5 in peripheral blood mononuclear cells. PLoS ONE.

[40] Liao K., Yan J., Mai K., Ai Q. (2015). Dietary olive and perilla oils affect liver mitochondrial DNA methylation in large yellow croakers. J. Nutr..

[41] Govindarajah V., Leung Y.K., Ying J., Gear R., Bornschein R.L., Medvedovic M., Ho S.M. (2016). In utero exposure of rats to high-fat diets perturbs gene expression profiles and cancer susceptibility of prepubertal mammary glands. J. Nutr. Biochem..

[42] García-Escobar E., Monastero R., García-Serrano S., Gómez-Zumaquero J.M., Lago-Sampedro A., Rubio-Martín E., Colomo N., Rodríguez-Pacheco F., Soriguer F., Rojo-Martínez G. (2017). Dietary fatty acids modulate adipocyte TNFα production via regulation of its DNA promoter methylation levels. J. Nutr. Biochem..

[43] Monastero R., García-Serrano S., Lago-Sampedro A., Rodríguez-Pacheco F., Colomo N., Morcillo S., Martín-Nuñez G.M., Gomez-Zumaquero J.M., García-Fuentes E., Soriguer F. (2017). Methylation patterns of Vegfb promoter are associated with gene and protein expression levels: The effects of dietary fatty acids. Eur. J. Nutr..

[44] Tezcan G., Tunca B., Demirci H., Bekar A., Taskapilioglu M.O., Kocaeli H., Egeli U., Cecener G., Tolunay S., Vatan O. (2017). Olea europaea leaf extract improves the efficacy of temozolomide therapy by inducing MGMT methylation and reducing P53 expression in glioblastoma. Nutr. Cancer.

[45] Arpón A., Milagro F.I., Razquin C., Corella D., Estruch R., Fitó M., Marti A., Martínez-González M.A., Ros E., Salas-Salvadó J. (2017). Impact of consuming extra-virgin olive oil or nuts within a Mediterranean diet on DNA methylation in peripheral white blood cells within the PREDIMED-Navarra randomized controlled trial: A role for dietary lipids. Nutrients.

[46] Corominas-Faja B., Cuyàs E., Lozano-Sánchez J., Cufí S., Verdura S., Fernández-Arroyo S., Borrás-Linares I., Martin-Castillo B., Martin Á.G., Lupu R. (2018). Extra-virgin olive oil contains a metabolo-epigenetic inhibitor of cancer stem cells. Carcinogenesis.

[47] Hunter D.J., James L., Hussey B., Wadley A.J., Lindley M.R., Mastana S.S. (2019). Impact of aerobic exercise and fatty acid supplementation on global and gene-specific DNA methylation. Epigenetics.

[48] Garcia-contreras C., Vazquez-Gomez M., Barbero A., Pesantez J.L., Zinellu A., Berlinguer F., Gonzalez-Añover P., Gonzalez J., Encinas T., Torres-Rovira L. (2019). Polyphenols and IUGR pregnancies: Effects of maternal hydroxytyrosol supplementation on placental gene expression and fetal antioxidant status, DNA-methylation and phenotype. Int. J. Mol. Sci..

[49] Oliveras-Ferraros C., Fernández-Arroyo S., Vazquez-Martin A., Lozano-Sánchez J., Cufí S., Joven J., Micol V., Fernández-Gutiérrez A., Segura-Carretero A., Menendez J.A. (2011). Crude phenolic extracts from extra virgin olive oil circumvent de novo breast cancer resistance to HER1/HER2-targeting drugs by inducing GADD45-sensed cellular stress, G2/M arrest and hyperacetylation of Histone H3. Int. J. Oncol..

[50] Luccarini I., Grossi C., Rigacci S., Coppi E., Pugliese A.M., Pantano D., la Marca G., Ed Dami T., Berti A., Stefani M. (2015). Oleuropein aglycone protects against pyroglutamylated-3 amyloid-ß toxicity: Biochemical, epigenetic and functional correlates. Neurobiol. Aging.

[51] Bonvino N.P., Ray N.B., Luu V.T., Liang J., Hung A., Karagiannis T.C. (2015). Exploration of mechanisms in nutriepigenomics: Identification of chromatin-modifying compounds from Olea europaea. Hell. J. Nucl. Med..

[52] Acevedo N., Frumento P., Harb H., Alashkar Alhamwe B., Johansson C., Eick L., Alm J., Renz H., Scheynius A., Potaczek D.P. (2019). Histone acetylation of immune regulatory genes in human placenta in association with maternal intake of olive oil and fish consumption. Int. J. Mol. Sci..

[53] Verdura S., Cuyàs E., Lozano-Sánchez J., Bastidas-Velez C., Llorach-Parés L., Fernández-Arroyo S., Hernández-Aguilera A., Joven J., Nonell-Canals A., Bosch-Barrera J. (2019). An olive oil phenolic is a new chemotype of mutant isocitrate dehydrogenase 1 (IDH1) inhibitors. Carcinogenesis.

[54] Bayat S., Mansoori Derakhshan S., Mansoori Derakhshan N., Shekari Khaniani M., Alivand M.R. (2019). Downregulation of HDAC2 and HDAC3 via oleuropein as a potent prevention and therapeutic agent in MCF-7 breast cancer cells. J. Cell. Biochem..

[55] Cuyàs E., Gumuzio J., Lozano-Sánchez J., Carreras D., Verdura S., Llorach-Parés L., Sanchez-Martinez M., Selga E., Pérez G.J., Scornik F.S. (2019). Extra virgin olive oil contains a phenolic inhibitor of the histone demethylase LSD1/KDM1A. Nutrients.

[56] Cuyàs E., Castillo D., Llorach-Parés L., Lozano-Sánchez J., Verdura S., Nonell-Canals A., Brunet J., Bosch-Barrera J., Joven J., Valdés R. (2019). Computational de-orphanization of the olive oil biophenol oleacein: Discovery of new metabolic and epigenetic targets. Food Chem. Toxicol..

[57] Mansouri N., Alivand M.R., Bayat S., Khaniani M.S., Derakhshan S.M. (2019). The hopeful anticancer role of oleuropein in breast cancer through histone deacetylase modulation. J. Cell. Biochem..

[58] Rodríguez-Miguel C., Moral R., Escrich R., Vela E., Solanas M., Escrich E. (2015). The role of dietary extra virgin olive oil and corn oil on the alteration of epigenetic patterns in the rat DMBA-induced breast cancer model. PLoS ONE.

[59] Bordoni L., Fedeli D., Fiorini D., Gabbianelli R. (2019). Extra virgin olive oil and Nigella sativa oil produced in central Italy: A comparison of the nutrigenomic effects of two Mediterranean oils in a low-grade inflammation model. Antioxidants.

[60] Di Francesco A., Falconi A., Di Germanio C., Micioni Di Bonaventura M.V., Costa A., Caramuta S., Del Carlo M., Compagnone D., Dainese E., Cifani C. (2015). Extravirgin olive oil upregulates CB_1_ tumor suppressor gene in human colon cancer cells and in rat colon via epigenetic mechanisms. J. Nutr. Biochem..

[61] Nanda N., Mahmood S., Bhatia A., Mahmood A., Dhawan D.K. (2019). Chemopreventive role of olive oil in colon carcinogenesis by targeting noncoding RNAs and methylation machinery. Int. J. Cancer.

[62] ArunSundar M., Shanmugarajan T.S., Ravichandiran V. (2018). 3,4-Dihydroxyphenylethanol assuages cognitive impulsivity in Alzheimer’s disease by attuning HPA-axis via differential crosstalk of Α7 NAChR with MicroRNA-124 and HDAC6. ACS Chem. Neurosci..

[63] Juli G., Oliverio M., Bellizzi D., Gallo Cantafio M.E., Grillone K., Passarino G., Colica C., Nardi M., Rossi M., Procopio A. (2019). Anti-tumor activity and epigenetic impact of the polyphenol oleacein in multiple myeloma. Cancers.

[64] Fraga M.F., Esteller M. (2005). Towards the human cancer epigenome: A first draft of histone modifications. Cell Cycle.

[65] Sanchez-Mut J.V., Gräff J. (2015). Epigenetic Alterations in Alzheimer’s Disease. Front. Behav. Neurosci..

[66] Adwan L., Zawia N.H. (2013). Epigenetics: A novel therapeutic approach for the treatment of Alzheimer’s disease. Pharmacol. Ther..

[67] Leri M., Scuto M., Ontario M.L., Calabrese V., Calabrese E.J., Bucciantini M., Stefani M. (2020). Healthy effects of plant polyphenols: Molecular mechanisms. Int. J. Mol. Sci..

[68] Adamo A., Sesé B., Boue S., Castaño J., Paramonov I., Barrero M.J., Izpisua Belmonte J.C. (2011). LSD1 regulates the balance between self-renewal and differentiation in human embryonic stem cells. Nat. Cell Biol..

[69] Lv T., Yuan D.M., Miao X., Lv Y., Zhan P., Shen X., Song Y. (2012). Over-Expression of LSD1 promotes proliferation, migration and invasion in non-small cell lung cancer. PLoS ONE.

[70] Serra G., Incani A., Serreli G., Porru L., Melis M.P., Tuberoso C.I.G., Rossin D., Biasi F., Deiana M. (2018). Olive oil polyphenols reduce oxysterols -induced redox imbalance and pro-inflammatory response in intestinal cells. Redox Biol..

[71] Lucas L., Russell A., Keast R. (2011). Molecular mechanisms of inflammation. Anti-inflammatory benefits of virgin olive oil and the phenolic compound oleocanthal. Curr. Pharm. Des..

[72] Rigacci S., Stefani M. (2016). Nutraceutical properties of olive oil polyphenols. An itinerary from cultured cells through animal models to humans. Int. J. Mol. Sci..

[73] Liberati A., Altman D.G., Tetzlaff J., Mulrow C., Gøtzsche P.C., Ioannidis J.P.A., Clarke M., Devereaux P.J., Kleijnen J., Moher D. (2009). The PRISMA statement for reporting systematic reviews and meta-analyses of studies that evaluate healthcare interventions: Explanation and elaboration. J. Clin. Epidemiol..

