# Supplementary material for: Epigenetic Modifications Induced by Olive Oil and Its Phenolic Compounds: A Systematic Review
Source: Molecules. 2021 Jan 7;26(2):273. doi: 10.3390/molecules26020273 (PMC7826507; doi:10.3390/molecules26020273)
Supplement: Supplementary file 1 [file molecules-26-00273-s001.pdf]

Table 1S. Characteristics of selected studies showing the effects of olive oil and/or its phenolic compounds on miRNAs (expression)

Reference	Compound<br>(Dose)	Model	Epigenetic Effects	Other effects<br>(Health related effects)
Tunca, 2012 [21]	*Olea europaea* leaf extract: OLE<br>(5-2000 µg/ml, 15% oleuropein)<br><br>Tested the effects in combination with TMZ[1]	In vitro:<br>-T98G human GBM[2] cell line	40 miRNAs were screened<br>OLE:<br>↑miRNA-584, miRNA-210, miRNA-219-1-3b, miRNA-181b, miRNA-145, miRNA-137<br>↓miRNA-24<br>=miRNA-153, let-7d<br>OLE + TMZ:<br>↑miRNA-181b, miRNA-153, miRNA-145, miRNA-137, let-7d	↓Proliferation<br>Synergistic effect with TMZ<br><br>**(Anti-cancer)**
Tezcan, 2014 [22]	*Olea europaea* leaf extract: OLE<br>(5-2000 µg/ml, 15% oleuropein)<br><br>Tested the effects in combination with TMZ	In vitro:<br>-T98G, U-138MG and U-187MG human GBM cell lines<br>-GBM stem-like cells (GSCs)	OLE in GSCs<br>↑miRNA-153, miRNA-137<br>=miRNA-181b, miRNA-145, Let-7d<br>OLE + TMZ in GSCs:<br>↑miRNA-153, miRNA-137, miRNA-181b, miRNA-145,<br>=Let-7d	↓Proliferation(U-138MG and U-187MG)<br>↑Apoptosis (T98G,U-138MG and U-187MG)<br>↓mRNA of: *TP53, OCT-4, SOX2, BCL2, c-myc*, *c-Met* (GSCs)<br>**(Anti-cancer)**
Casas-Agustench, 2015, [23]	Olive oil as a source of n-9 fatty acids	In vivo:<br>-Female Sprague-Dawley rats	↑miRNA-215, miRNA-10b, miRNA-26, miRNA-377-3p, miRNA-21, miRNA-192	No effect on 88 genes related to insulin signaling<br>**(Insulin sensitivity)**
Di Francesco, 2015 [60]	-EVOO[3]<br>(In vitro: 100 ppm containing 320 mg of total phenols/kg)<br>(In vivo: gavage, 250 µl/300 g)	In vitro:<br>-CaCo-2 human colorectal cancer cells<br>In vivo:<br>-Female Sprague-Dawley rats	In vivo:<br>↓miRNA-23, miRNA-301a<br>=miRNA-27a, miRNA-29a	↑CB1[4] (protein)<br>↑mRNA of: *CNR1*<br>↓Proliferation (Caco-2 cells)<br>**(Anti-cancer)**

Tomé-Carneiro, 2016 [24]	Hydroxytyrosol (HT)<br>(0.03 % HT in diet, long term)<br>(Gavage, 15mg of HT, acute)<br>(in vitro: 10 μM)<br>(Human: 25 mg/d)	In vitro:<br>-CaCo-2, InEpCells[5], mouse primary intestinal organoids or "miniguts"<br>In vivo:<br>-C57BL/6mice (8 weeks)<br>Human trial:<br>-PBMC	In vitro:<br>CaCo2: ↓miRNA-196b-3p<br>InEpCells: ↑miRNA-483-3p, miRNA-1247-5p<br>Miniguts: ↑miRNA-193a-5p, miRNA-1247-5p<br>In vivo: 752 miRNAs screened<br>↑miRNA-483-3p, miRNA-135a-1-3p, miRNA-1982-5p miRNA-196b-3p, miRNA-1898<br>↓miRNA-346- 5p<br>Human:<br>↑miRNA-193a-5p in PBMCs	↑plasma, liver and intestine triacylglycerol (mice)<br>mRNA microarrays:<br>↓332 genes including Cd36, Cyp4a10, Gsta3, Hsd3b2, Paqr7, Pdk4 and Slc35e3<br>↑342 genes including Car2, Crym, Cxcl13, Dusp6, Hsph1 and Il33<br>**(Lipid metabolism and Anti-CVD)**
D'Amore, 2016 [25]	EVOO (50 mL, 4h)<br>(High phenols: 491 mg/kg)<br>(Low phenols: 270 mg/Kg)	Human trial:<br>-Healthy subjects (n=12)<br>-MetS[6] subjects (n=12)	↓8 miRNA:<br>miRNA-146b-5p, miRNA-19a-3p, miRNA-181b-5p, miRNA-107, miRNA-769-5p, miRNA-192-5p<br>↑6 miRNA:<br>miRNA-23b-3p, miRNA-519b-3p	↓glucose, insulin, HOMA-IR<br>↓1062 genes healthy<br>↑1376 genes healthy<br>↓551 genes MetS<br>↑403 genes MetS<br>**(Anti-inflammatory, Anti-CVD and Anti-cancer)**
Xu, 2017[26]	Oleuropein<br>(In vitro: 200 μM)<br>(In vivo:drinking water, 1% w/v)	In vitro:<br>-NPC[7]HNE1 and HONE1 cell lines<br>In vivo:<br>-xenograft NPC mouse model	↑22 different miRNAs<br>↑miRNA-519d	↑Radio-sensitivity<br>↓mRNA and protein levels ofHIF1α[8], p53 and PDRG1[9]<br><br>**(Anti-cancer)**
D'Adamo, 2017 [27]	Hydroxytyrosol<br>100 μM	In vitro:<br>-Human primary and C-28/I2 chondrocytes	↓Up-regulation of miRNA-9 induced by H_2O_2	↑Down-regulation of SIRT-1 induced by H_2O_2<br>**(Anti-osteoarthritis and anti-oxidant)**
Bigagli, 2017 [28]	Hydryxytyrosol, Oleuropein<br>(10 μM)	In vitro:<br>-RAW264.7 macrophage cells stimulated with LPS	↓ miRNA-146a	↓Oxidative burst<br>↓NO and PGE2<br>↓CD11b expression<br>↑Nrf2 translocation

				**(Anti-inflammatory)**
Luceri, 2017 [29]	EVOO<br>(10 g/100 g of diet)<br>(High phenols: 718.8 mg/kg)<br>(Low phenols: 9.3 mg/Kg)	In vivo:<br>-Middle-aged C57Bl/6J mice 6 months	↑miRNA-484, miRNA-27, miRNA-137, miRNA-30, miRNA-34, miRNA-124	↑Motor and cognitive behavior<br>↑Notch1, BMPs, NGFR GLP1R, CRTC3<br>**(Anti-aging)**
Xing, 2017 [30]	Oleuropein<br>(In vitro: 200 μM)<br>(In vivo: drinking water, 1% w/v)	In vitro:<br>-Caov3 and Skov3 ovarian cancer cell lines<br>In vivo:<br>-xenograft ovarian cancer mouse model	↑10 different miRNAs<br>↑miRNA-299	↑Radio-sensitivity<br>↓mRNA of HPSE1<br>↓HIF1α<br><br>**(Anti-cancer)**
Abtin, 2018 [31]	Oleuropein<br>(600-1000μg/mL)	In vitro:<br>-Human MCF-7 breast cancer cell line	↓miRNA-21, miRNA-155	↑Apoptosis<br>↓Viability, Migration<br>↑APAF-1, PTEN, TP53INP-1, and FADD genes<br>**(Anti-cancer)**
ArunSundar, 2018 [62]	Hydroxytyrosol<br>(In vitro: 100 μM)<br>(In vivo: not reported)	In vivo:<br>-Animal model: Alzheimer's Disease induced in mouse by oA42i[10]<br>-Organotypic hippocampal slice cultures (OHSCs)	↑ miRNA-124	↑Cognitive impulsivity<br>↓Anxiety-like behavior<br>↓HDAC6, HSP90<br><br>**(Anti-Alzheimer's Disease)**
Nanda, 2019 [61]	EVOO<br>(Thrice a week, 1 g/kg body weight, gavage)	In vivo:<br>-Sprague Dawley rats exposed to DHM[11] (model of colon carcinogenesis)	↑miRNA-143 and miRNA-145 expression<br>↓Methylation of promoter region of miR-143 and miR-145	↑body weights<br>↓tumor or polyp incidence, multiplicity and size<br>↓mRNA and protein expression of NF-κB, VEGF, MMP-9<br>↑mRNA and protein expression of caspase-3 and caspase-9<br>(Anti-cancer and Anti-inflammatory)

Juli, 2019 [63]	Oleacein (3,4-DHPEA-EDA)<br>(2,5-10 μM)	In vitro:<br>-NCI-H929, RPMI-8226, U266, MM1s and JJN3 MM[12] cell lines	↑miRNA-29b and miRNA-22 in JJN3	↓Viability and clonogenicity of MM cells<br>=viability of normal PBMC<br>↓Cell cycle progression<br>↑Apoptosis<br>↓ Sp1 protein expression<br>↑in vitro anti-MM activity of the proteasome inhibitor carfilzomib<br>**(Anti-cancer)**
Benincasa, 2019 [32]	Hydroxytyrosol oleate<br>(5 μM)	In vitro:<br>-HaCat human immortalized keratinocyte cell line	↑miRNA-34a, miRNA-21, miRNA-29a	↑Viability<br>↓Intracellular ROS formation<br>↓SOD and GST enzyme activity<br>↓MDA<br>**(Anti-oxidant)**
Lopez De Las Hazas, 2019 [33]	Hydroxytyrosol<br>(0.03 % HT in the Diet)	In vivo:<br>-Mice liver samples obtained from a previous study [24]	247 different miRNAs detected in mouse liver samples<br>↑miRNA-802-5p, miRNA-30a-5p, miRNA-146b-5p<br>↓miRNA-423-3p	Expression of several genes and proteins<br>↑Fgf21 and Rora<br>**(Lipid metabolism and Anti-CVD)**
Tezcan, 2019 [34]	Oleuropein<br>(277.5 and 555 μM)<br><br>Tested the effects in combination with TMZ	In vitro:<br>-T98G human GBM cell line	↑miRNA-181b, miRNA-137 and Let-7d<br>=miRNA-153	↓Viability/proliferation<br><br>**(Anti-cancer)**
Scoditti, 2019 [35]	Hydroxytyrosol<br>(1-10 μM)	In vitro:<br>-Human SGBS[13] adipocytes stimulated with TNF-α	↓ upregulation of miRNA-34a, miRNA-155 induced by TNF-α<br>↑let-7c expression level decreased by TNF-α	↓TNF-α induced upregulation of mRNA of: MCP-1, CXCL-10, M-CSF, IL-1, VEGF, COX-2, MMP-2, IL-6, PAI-1, ICAM-1, SOD-1, GPX-1<br>↑ TNF-α induced downregulation of mRNA of: eNOS, PGC-1α, GLUT-4.

				↓ NFkB activation<br>**(Anti-inflammatory)**
D'Adamo, 2019 [36]	Hydroxytyrosol<br>(100 μM)	In vitro:<br>-Human primary and C-28/I2 chondrocytes	Restores the methylation of promoters of miRNA-9 decreased by H_2O_2	**(Anti-osteoarthritis and anti-oxidant)**
Carpi, 2019 [37]	Oleocanthal (*p*-DHPEA-EDA)<br>Oleacein (3,4-DHPEA-EDA)<br>25μM	In vitro:<br>-Human SGBS adipocytes stimulated with TNF-α	Counteract the TNF-α effects in cells and exosomes:<br>↓ miRNA-34a-5p and miRNA-155-5p<br>↑let-7c-5p	=Cell viability<br>↓TNF-α induced upregulation of mRNA of: IL-1β, COX-2, VEGF/KDR, MMP-2, NADPH oxidase, SOD, GPX, MCP-1, CXCL-10, MCS-F<br>↑expression of the anti-inflammatory/metabolic effector PPAR<br>↓ NFkB activation<br>**(Anti-inflammatory)**
Terzuoli, 2020 [38]	Hydroxytyrosol-3-O-sulphate<br>(major HT plasma metabolite, 10 μM)	In vitro:<br>-Human umbilical endothelial cells (HUVEC)<br>-Human retinal endothelial cells (HREC)<br>(stimulated withIL-1β)	↑*let-7* miRNA decreased by IL-1β	↓ IL-1β induced endothelial-to-mesenchymal transition (EndMT)<br>↑CD31, FGFR1 (protein)<br>↓α-SMA, vimentin (protein)<br>↓TGF-β signaling<br>↓nuclear translocation of SMAD2/3<br>↓ACTA2, ZEB2, SNAI1, VIM, NOTCH3, CNN1, MMP2, MMP9 (mRNA)<br>↑ CD31, FGFR1 (mRNA)<br>**(Anti-inflammatory)**

[1]Temozolomide, [2]Glioblastoma multiforme, [3]Extra virgin olive oil, [4]Type 1 cannabinoid receptor,[5]Human primary epithelial intestinal cells,[6]Metabolic syndrome[7]Nasopharyngeal carcinoma,[8]Hypoxia-inducible factor-1α,[9]DNA damage-regulated protein, [10]Soluble oligomeric amyloid β_{1-42} plus ibotenic acid, [11]1,2-dimethylhydrazine,[12]Multiple myeloma,[13]Simpson-Golabi-Behmel syndrome.

Table S2. Characteristics of selected studies showing the effects of olive oil and/or its phenolic compounds on DNA methylation

Ref.	Compound (dose)	Model	Epigenetic Effects	Other effects<br>Health related effects
Hoile, 2014 [39]	-Olive oil<br>(4 g daily, “1 g capsules”)<br>-n-3 LC-PUFA	Human trial:<br>-29 Chronic kidney disease patients, 8 weeks<br>-Peripheral blood mononuclear cells	DNA methylation of CpGs in 5’ regulatory regions of:<br>↑FADS2<br>= FADS1, ELOVL2<br>↑ELOVL5 in some regions and ↓in others	↓mRNA of: *FADS2, FADS1, ELOVL5* (only female), *ELOVL2*<br><br>**(PUFA metabolism)**
Rodríguez-Miguel, 2015 [58]	-Low fat<br>-Corn oil (CO)<br>-EVOO[1]<br>(17% in the diet)	In vivo:<br>-DMBA[2]-Induced Breast Cancer in rats	↑Global DNA methylation<br>↓promoter methylation of *RASSF1A* and *TIMP3 vs.* CO<br>↓mRNA of *DNMT1, DNMT3a DNMT3b vs.* CO<br>↓DNMT activity vs. CO	Weak tumor enhancing effect<br>↓mRNA of: *RASSF1A, TIMP3,*<br>**(Anti-cancer)**
Di Francesco, 2015 [60]	-ROO[3]<br>-EVOO<br>(In vitro: 100 ppm containing 320 mg of total phenols/kg)<br>(In vivo: gavage, 250 µl/300 g)<br>-OPE[4] (50 µM)<br>-Hydroxytyrosol (50 µM)	In vitro:<br>-CaCo-2 human colorectal cancer cells<br>In vivo:<br>-Female Sprague-Dawley rats<br>(Single dose and 10 days of EVOO)	*CNR1* gene promoter DNA methylation:<br>=ROO<br>↓EVOO (in vitro and in vivo)<br>↓OPE<br>↓HT	CB1[5] (mRNA and protein expression):<br>↑EVOO (in vitro and in vivo)<br>↑OPE<br>↑HT<br>↓Proliferation (OPE, HT)<br>**(Anti-cancer)**
Liao, 2015 [40]	Fish oil (FO as control)<br>Olive oil<br>(80 g/kg of diet)	In vivo:<br>-Yellow croakers (*Larimichthyscrocea*)<br>70 days	Mitochondrial DNA methylation<br>↑*TR* and *ND4L*<br>↓*RNR1*<br>=*D-loop*<br>mRNA and protein:<br>↓DNMT1, DNMT3B<br>↑DNMT3A, HIF1a	↑Liver lipid<br>↑Hydroxyl radical scavenging activity<br>**(Anti-oxidant)**

Govindarajah, 2016 [41]	Butter fat<br>Safflower oil<br>Olive oil<br>(40% fat from olive oil)	In vivo:<br>-DMBA-Induced Breast Cancer in rats<br>(prenatal exposure, cancer in prepubertal mammary glands)	↑ mRNA expression of *DNMT3a*, *Mbd1*	=Tumor volume, latency, multiplicity<br>↓mRNA of: *Lrrn1, Nf1, Dbf4, Cadm4,*<br>↑mRNA of: *Tmem45b, Btn1a1*<br>**(Anti-cancer)**
Garcìa-Escobar, 2017 [42]	Coconut oil (CO)<br>Sunflower oil (SO)<br>Olive oil<br>(10% fat from oils)	In vivo:<br>-Sprague-Dawley rats<br>-Adipocytes	TNFα promoter methylation<br>↓ versus SO<br>↑ versus CO	↓mRNA of TNFα versus CO<br>↓release of TNFα from adipocytes versus CO<br>**(Anti-inflammatory)**
Monastero, 2017 [43]	Coconut oil (CO)<br>Sunflower oil (SO)<br>Olive oil<br>(10% fat from oils)	In vivo:<br>-Sprague–Dawley rats	↑VEGF promoter methylation versus CO	↓ mRNA and protein of VEGFB versus CO<br><br>**(Anti-obesity)**
Tezcan, 2017 [44]	*Olea europaea* leaf extract:<br>OLE (5-2000 μg/ml, 15% oleuropein)<br><br>Tested the effects in combination with TMZ	In vitro:<br>-Human primary GBM[6]tumor cells	↑ MGMT promoter methylation	↓Proliferation (with TMZ)<br>↑DNA damage (with TMZ)<br>↓p53 (protein)<br>**(Anti-cancer)**
Arpón, 2018 [45]	MedDiet + EVOO (39.6-66.4 g/day)<br>MedDiet + nuts (MN)<br>Low fat (RF)	Human trial:<br>-36 subjects, 5 years<br>-Peripheral white blood cells	223 CpGs screened<br>↓ DNA methylation of cg17071192–GNAS/GNASAS versus both MN and RF	↓Diabetes<br>↓Hypercholesterolemia<br>↓Arterial hypertension<br>**(Anti-CVD)**
Corominas-Faja, 2018 [46]	Oleacein (3,4-DHPEA-EDA)<br>(0,1-20 μM)	In vitro:<br>-SUM-159 Breast Cancer cells (CSC)<br>-MCF-7<br>In vivo:	↓DNMT1, 3A/3L and 3B/3Lenzyme activity in nuclear extract<br><br>Act as SAM competitive inhibitor	Antistemness properties:<br>↓ALDH^{+} cells<br>↓mammospheres formation<br>↓ tumorigenicityin vivo<br>↓mTOR activity<br>↓44 genes

		-Tumor xenograft and orthotopic transplant studies		↑116 genes<br>**(Anti-cancer)**
Hunter, 2019 [47]	-EVOO<br>(6 g daily, “capsules”)<br>-n-3 PUFA	Human trial:<br>-8 subjects (trained male cyclists), 4 weeks<br>-Whole blood isolated from	=Global DNA methylation<br>=mRNA expression of *DNMT3a, DNMT3b*<br>↓mRNA expression of *DNMT1*<br>=Methylation of PPARGC1A<br>↓Methylation of IL6<br>=Methylation of TNF	=mRNA expression of *IL6, TNF, PPARGC1A*<br><br>**(Anti-inflammatory)**
Nanda, 2019 [61]	EVOO<br>(Thrice a week, 1 g/kg body weight, gavage)	In vivo:<br>-Sprague Dawley rats exposed to DHM[7] (model of colon carcinogenesis)	↑methylation of promoter region of NF-κB, VEGF, MMP-9,<br>↓methylation promoter region of miR-143, miR-145, caspase-3, caspase-9	↑body weights<br>↓tumor or polyp incidence, multiplicity and size<br>↓mRNA and protein expression of NF-κB, VEGF, MMP-9<br>↑mRNA and protein expression of caspase-3 and caspase-9<br>**(Anti-cancer and Anti-inflammatory)**
Garcia-Contreras, 2019 [48]	Hydroxytyrosol<br>(1.5 mg/kg of feed per day)	In vivo:<br>-Sows (maternal supplementation), 65 days<br>-Blood and plasma (maternal and fetal)	↑Global DNA methylation of fetus	Undetectable Plasma hydroxytyrosol concentrations<br>↑total antioxidant capacity (TEAC)<br>↓ body and visceral weights<br>↑ diameter of the descending aorta;<br>↓ Parameters for glycemic index (glucose, fructosamine) and lipid profile<br>=SOD1, CAT, HIF1A, VEGFA, NOS2, IGF1 and UCP2 (mRNA)

				**(Anti-intrauterine growth restriction)**
Juli, 2019 [63]	Oleacein (3,4-DHPEA-EDA) (2,5-10 µM)	In vitro:<br>-NCI-H929, RPMI-8226, U266, MM1s and JJN3 MM[8] cell lines	=Global DNA methylation<br>=mRNA and protein expression of: DNMT1, DNMT3A, DNMT3B	↓Viability and clonogenicity of MM cells<br>=viability of normal PBMC<br>↓Cell cycle progression<br>↑Apoptosis<br>↓ Sp1 protein expression<br>↑in vitro anti-MM activity of the proteasome inhibitor carfilzomib<br>**(Anti-cancer)**
Bordoni, 2019 [59]	-EVOO<br>(volumes dissolved in DMSO not reported)<br>-*Nigella sativa* oil (NG)	In vitro:<br>-THP-1 (stimulated with PMA and LPS)	↓mRNA of DNMT3A, DNMT3B<br>=DNMT1<br>↑Global DNA methylation versus NG<br>↓TET2<br>=TET1, TET3	=Cell viability<br>↓IL-6<br>↓IL-1β<br>↓MCP-1<br>↓MtDNA copy number<br>↓Membrane fluidity<br>= (GSH/GSSG) Ratio<br><br>**(Anti-inflammatory)**

[1]Extra virgin olive oil, [2]Dimethylbenz(a)anthracene, [3]Rectified olive oil, [4]Olive oil phenolic extract, [5]Type 1 cannabinoid receptor,[6]Glioblastoma multiforme,[7]1,2-dimethylhydrazine, [8]Multiple myeloma

Table 3. Characteristics of selected studies showing the effects of olive oil and/or its phenolic compounds on Histone modifications

**Ref.**	**Compound (dose)**	**Model**	**Epigenetic Effects**	**Other effects**<br>**Health related effects**
Oliveras-Ferraros, 2011 [49]	-EVOO[1] phenolic extract from 14 monovarietals (0%, 0.0001, 0.001, 0.01, 0.05 and 0.1 % v/v)	In vitro:<br>-JIMT-1 human breast cancer cell line	↑Acetylation of Histone H3 at Lysine 18	↓Cell viability<br>↓Cell cycle (arrest at the G2/M phase)<br>= AKT<br>↑Stat3<br>↑p38MAPK<br>↑MEK1<br>↑NF-kB phosphorylation status<br>**(Anti-cancer)**
Rodríguez-Miguel, 2015 [58]	-Low fat<br>-Corn oil (CO)<br>-EVOO<br>(17% in the diet)	In vivo:<br>-DMBA[2]-Induced Breast Cancer in rats	↓H4K20me3 in tumor tissue<br>↓H4K16ac in normal and tumor tissue<br>= H3K4me2 and H3K27me3 in normal and tumor tissue	Weak tumor enhancing effect<br>↓mRNA of: *RASSF1A, TIMP3,*<br>**(Anti-cancer)**
Luccarini, 2015 [50]	-Oleuropein aglicone (OLE, 100μM)<br>-OLE diet (50 mg/kg of diet)	In vivo:<br>-TgCRND8[3] AD mouse model<br>In vitro:<br>-pE3-Aβ[4] + OLE	↑H3K9 and H4K5 acetylation<br>↓HDAC2 expression	pE3-Aβaggregation modified<br>↓pE3-Aβ citotoxicity<br>↓Glutaminyl cyclase (QC)<br>↑Autophagy<br>↓ dysfunctions of transgene-induced long-term potentiation(LTP) in the hippocampal area<br>↑OLE metabolites in the brain<br>**(Anti-Alzheimer's Disease)**
Bonvino, 2015 [51]	-Hydroxytyrosol<br>(0-100μM)	In vitro:	↑HDAC6<br>↓KDM1A (LSD1)	= acetylated α tubulin

	A commercial topical antimicrobial spray containing the Olivamine 10 ® formulation, which includes a hydroxytyrosol containing olive extract	-purified enzyme LSD1 + hydroxytyrosol<br>Ex vivo:<br>-Tissue sections obtained from the peri wound of a diabetic ulcer patient		**(Anti diabetes adverse effects)**
ArunSundar, 2018 [62]	Hydroxytyrosol<br>(In vitro: 100 μM)<br>(In vivo: not reported)	In vivo:<br>-Animal model: Alzheimer's Disease induced in mouse by oA42i[5]<br>-Organotypic hippocampal slice cultures (OHSCs)	↓ HDAC6	↓Cognitive impulsivity<br>↓Anxiety-like behavior<br>↓ HSP90<br>**(Anti-Alzheimer's Disease)**
Acevedo, 2019 [52]	-Olive oil in the diet	In vivo:<br>-Human Placenta (ALADDIN[6] cohort)<br>Cross-sectional study	↑H3 acetylation at FOXP3, IL10RA and IL7R promoters	**(Immune system)**
Verdura, 2019 [53]	-Oleacein (Decarboxymethyl oleuropein aglycone (DOA) (3,4-DHPEA-EDA) (0.01-100μM)	In vitro:<br>-purified enzyme IDH1 + oleacein<br>-MCF10/HCT116 cells expressing R132H IDH1 mutant and their parental cell lines	↓ (H3K9me3)	↓ R132H IDH1<br>↓ 2HG<br>↑PD-L1<br>↓colony formation<br>**(Anti-cancer)**
Bayat, 2019 [54]	-Oleuropein glucoside (150-2400μg/ml)	In vitro:<br>MCF7 human breast cancer cell line	↓HDAC2 e HDAC3 mRNA expression	↓cell viability<br>↑ cell apoptosis<br>↓cell migration<br>**(Anti-cancer)**
Juli, 2019 [63]	-Oleacein (3,4-DHPEA-EDA) (2,5-10 μM)	In vitro:	↑ H3 and H4 acetylation<br>↓I/II HDACs (HDAC1/2/3/4/6)	↓Viability and clonogenicity of MM cells<br>=viability of normal PBMC

		-NCI-H929, RPMI-8226, U266, MM1s and JJN3 MM[7] cell lines	=HDAC activity	↓Cell cycle progression<br>↑Apoptosis<br>↓ Sp1 protein expression<br>↑in vitro anti-MM activity of the proteasome inhibitor carfilzomib<br>**(Anti-cancer)**
Cuyàs, 2019 [55]	-Oleacein<br>(3,4-DHPEA-EDA)<br>(0,01-100µM)	In vitro:<br>- Purified enzyme (KDM1A) + oleacein<br>-BT-474 and MCF7 breast cancer cell lines	↓KDM1A (LSD1)	↓SOX2<br>**(Anti-cancer)**
Cuyas, 2019 [56]	-Oleacein<br>(3,4-DHPEA-EDA)<br>(1-100µM)	In vitro:<br>-Purified enzyme + oleacein<br>-MCF-7 and BT-474 breast cancer cell lines	↓KDM6A<br>↓DOT1L	↓NNMT<br>↓ACLY<br>**(Anti-cancer)**
Mansouri, 2019 [57]	-Oleuropein glucoside<br>150-2400µg/ml	In vitro:<br>-MCF-7 breast cancer cell line	↓mRNA HDAC4	↓cell Viability<br>↑ cell apoptosis<br>↓ cell migration<br>**(Anti-cancer)**
Bordoni, 2019 [59]	-EVOO<br>(volumes dissolved in DMSO not reported)<br>-*Nigella sativa* oil (NG)<br><br><u>No phenols</u>	In vitro:<br>-THP-1 (stimulated with PMA and LPS)	↓HDAC1, HDAC3	=cell viability<br>↓IL-6<br>↓IL-1β<br>↓MCP-1<br>↓MtDNA copy number<br>↓Membrane fluidity<br>= (GSH/GSSG) Ratio<br>**(Anti-inflammatory)**

[1]Extra virgin olive oil, [2]Dimethylbenz(a)anthracene, [3]Transgenic hemizygous CRND8 mice; [4] pE3-Aβ pyroglutamate-amyloid-β; [5]oligomeric amyloid β_{1-42} plus ibotenic acid; [6]Assessment of Lifestyle and Allergic Disease During INfancy (ALADDIN) cohort; [7]Multiple myeloma
